# P-2062. COVID-19 XBB.1.5-adapted Vaccine Uptake in Immunocompromised Individuals Using Tokenized State Vaccine Registries

**DOI:** 10.1093/ofid/ofae631.2218

**Published:** 2025-01-29

**Authors:** Matthew A Brouillette, Farid L Khan, Santiago M C Lopez, Kathleen M Andersen, Tiange Yu, Benjamin T Carter, John M McLaughlin, Leah McGrath

**Affiliations:** Pfizer Inc., New York, New York; Pfizer, Collegeville, Pennsylvania; Pfizer Inc, Collegeville, Pennsylvania; Pfizer Inc., New York, New York; Genesis Research Group, Hoboken, New Jersey; Genesis Research Group, Hoboken, New Jersey; Pfizer, Collegeville, Pennsylvania; Pfizer, Collegeville, Pennsylvania

## Abstract

**Background:**

Immunocompromised individuals experience a disproportionate burden of COVID-19-associated hospitalization and death. COVID-19 vaccine uptake has declined over time. We evaluated XBB.1.5-adapted vaccine uptake, regardless of brand, during the 2023-2024 season among immunocompromised persons using data from two large state vaccine registries.

**Figure:**
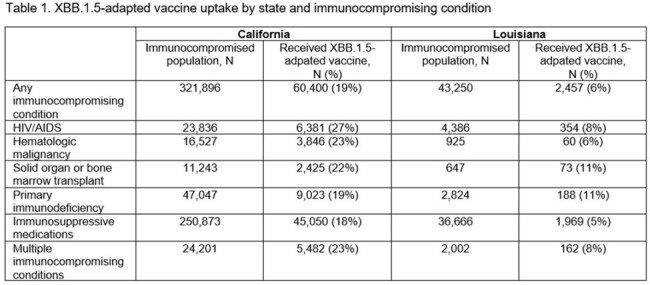

**Methods:**

Individuals with continuous pharmacy and medical enrollment (≥6 months for children < 18 years, ≥12 months for adults ≥18 years) in insurance plans contributing to HealthVerity and residency in either California or Louisiana as of September 11, 2023 (the date XBB.1.5-adapted vaccines were authorized or approved) were selected. A prior dose of any COVID-19 vaccine was required. Persons were defined as immunocompromised if they had one or more of the following conditions as defined by the Centers for Disease Control and Prevention: HIV/AIDS, hematologic malignancy, solid organ or bone marrow transplant, primary immunodeficiency, or recent use of immunosuppressive medications. XBB.1.5-adapted vaccine uptake was assessed using de-identified records from state vaccine registries in California and Louisiana that were linked via tokenization with claims data.

**Results:**

A total of 321,896 California residents (median age 55 years; 57% female) and 43,250 Louisiana residents (median age 45 years; 64% female) with at least one immunocompromising condition were identified. In both states, the most common immunocompromising condition was use of immunosuppressive medications (78% California and 85% Louisiana). By the end of March 2024, 19% and 6% of immunocompromised individuals in California and Louisiana, respectively, received an XBB.1.5-adapted vaccine. Uptake was generally similar regardless of immunocompromising condition (Table 1).

**Conclusion:**

During the 2023-2024 season, COVID-19 vaccine uptake in immunocompromised persons was low, with fewer than 20% of this high-risk population receiving the XBB.1.5-adapted vaccine. Enhanced efforts to increase COVID-19 vaccine uptake in this vulnerable population are urgently needed.

**Disclosures:**

Matthew A. Brouillette, MPH, Pfizer Inc.: Employee|Pfizer Inc.: Stocks/Bonds (Public Company) Farid L. Khan, MPH, Pfizer Inc.: Employee|Pfizer Inc.: Stocks/Bonds (Public Company) Santiago M.C. Lopez, MD, Pfizer Inc.: Employee|Pfizer Inc.: Stocks/Bonds (Public Company) Kathleen M. Andersen, PhD MSc, Pfizer Inc.: Employee|Pfizer Inc.: Stocks/Bonds (Public Company) Tiange Yu, MS, Pfizer Inc.: Employee of Genesis Research Group which has received consulting fees from Pfizer Inc. Benjamin T. Carter, PhD, Pfizer Inc.: Employee of Genesis Research Group which has received consulting fees from Pfizer Inc. John M. McLaughlin, PhD, Pfizer: Employee|Pfizer: Stocks/Bonds (Public Company) Leah McGrath, PhD, Pfizer Inc.: Employee|Pfizer Inc.: Stocks/Bonds (Public Company)

